# Amelioration of myocardial ischemia/reperfusion injury in diabetes: A narrative review of the mechanisms and clinical applications of dexmedetomidine

**DOI:** 10.3389/fphar.2022.949754

**Published:** 2022-08-31

**Authors:** Meng Sun, Rong Wang, Rui Xia, Zhengyuan Xia, Zhilin Wu, Tingting Wang

**Affiliations:** ^1^ Department of Anesthesiology, Union Hospital, Tongji Medical College, Huazhong University of Science and Technology, Wuhan, China; ^2^ Institute of Anesthesia and Critical Care Medicine, Union Hospital, Tongji Medical College, Huazhong University of Science and Technology, Wuhan, China; ^3^ State Key Laboratory of Pharmaceutical Biotechnology, The University of Hong Kong, Hong Kong, China; ^4^ Department of Anesthesiology, Affiliated Hospital of Guangdong Medical University, Zhanjiang, China

**Keywords:** cardioprotection, ischemia-reperfusion, oxidative stress, autophagy, inflammation, apoptosis, dexmedetomidine

## Abstract

Mechanisms contributing to the pathogenesis of myocardial ischemia-reperfusion (I/R) injury are complex and multifactorial. Many strategies have been developed to ameliorate myocardial I/R injuries based on these mechanisms. However, the cardioprotective effects of these strategies appear to diminish in diabetic states. Diabetes weakens myocardial responses to therapies by disrupting intracellular signaling pathways which may be responsible for enhancing cellular resistance to damage. Intriguingly, it was found that Dexmedetomidine (DEX), a potent and selective α2-adrenergic agonist, appears to have the property to reverse diabetes-related inhibition of most intervention-mediated myocardial protection and exert a protective effect. Several mechanisms were revealed to be involved in DEX’s protection in diabetic rodent myocardial I/R models, including PI3K/Akt and associated GSK-3β pathway stimulation, endoplasmic reticulum stress (ERS) alleviation, and apoptosis inhibition. In addition, DEX could attenuate diabetic myocardial I/R injury by up-regulating autophagy, reducing ROS production, and inhibiting the inflammatory response through HMGB1 pathways. The regulation of autonomic nervous function also appeared to be involved in the protective mechanisms of DEX. In the present review, the evidence and underlying mechanisms of DEX in ameliorating myocardial I/R injury in diabetes are summarized, and the potential of DEX for the treatment/prevention of myocardial I/R injury in diabetic patients is discussed.

## 1 Introduction

The prevalence of diabetes has rapidly reached an epidemic level globally, shaping the disease into one of the 21st-century healthcare challenges and posing an incredibly high economic burden to the whole world (Zimmet et al., 2014; Schmidt, 2018; Berezin and Berezin, 2019). Coronary occlusions are more likely to occur and the heart is more sensitive to ischemia-reperfusion (I/R) injury in diabetic patients than in non-diabetic patients (Sasso et al., 2011; Henning, 2018). The incidence of cardiovascular disease in adults with diabetes is two to three times higher than those without diabetes (Sarwar et al., 2010; Henning, 2018; Strain and Paldánius, 2018). One possible reason is that the diabetic patients share several pathological features including inflammation and oxidative stress (Fisher, 1999; Alegria et al., 2007; Marso et al., 2007; Miki et al., 2012; Lejay et al., 2016; Russo et al., 2017; Zhao et al., 2017). Additionally, cardioprotective interventions such as ischemic preconditioning and postconditioning that are effective in nondiabetic subjects, are largely ineffective in diabetes (Keating, 2015; Russell et al., 2019). Therefore, it is extremely important to identify new pharmacological targets for the prevention and treatment of diabetic myocardial I/R injury.

DEX is a potent α-2 adrenergic receptor agonist that has sedative, analgesic, anxiolytic and opioid-sparing properties (Ebert et al., 2000; Venn and Grounds, 2001; Panzer et al., 2009; Keating, 2015). For its low risk of respiration inhibition and unique property of organ protection, the application of DEX is gaining popularity (Belleville et al., 1992; Venn et al., 2000). Plenty of evidence proved that the administration of DEX could protect the intestine (Sun et al., 2015; Zhang et al., 2020a), heart (Zhang et al., 2020b; Wu et al., 2020), kidney (Li et al., 2018), lung (Liang et al., 2019), and liver (Sahin et al., 2013; Lim et al., 2021) against I/R injury through “pharmacological preconditioning” or “pharmacological postconditioning” (Cai et al., 2014). Although most interventions failed to confer protection against I/R injury in diabetic states, it is interesting to note that DEX could ameliorate diabetic I/R injuries of various organs in animal models (Arslan et al., 2012; Kip et al., 2015; Yeda et al., 2017; Liu C. Y. et al., 2018; Castillo et al., 2019; Chen et al., 2019; Hou et al., 2020). Thus, it is of great importance to elucidate the biological function and the associated molecular mechanism of DEX under diabetic conditions, which will be useful for new pharmaceutical target finding and drug development against I/R injury in diabetes. Here, in this paper, the mechanisms of myocardial I/R injury and current strategies are reviewed and the therapeutic value of DEX and its molecular mechanisms in the treatment of diabetic myocardial I/R injury are elaborated.

## 2 Basic mechanisms of myocardial I/R injury and current strategies of protective interventions

The restoration of blood supply to the ischemic myocardium after myocardial I/R paradoxically leads to more intense cellular damage, with complex, diverse and highly integrated pathogenesis (Frank et al., 2012). During ischemia, hydrogen ions accumulate in large quantities intracellularly (Frank et al., 2012). When myocardial blood supply is restored, intracellular pH rapidly returns to its physiological state, disturbing the ions exchange of sodium and calcium, and causing an increase in mitochondrial calcium ions (Yellon and Hausenloy, 2007; Frank et al., 2012). The calcium ions overload activates calpain pathway and contributes to cell death (Potz et al., 2016; Valikeserlis et al., 2021). The mitochondrial permeability transition pore (mPTP) would open in response to mitochondrial calcium ions overload, oxidative stress, and the restoration of a physiological pH (Zhang M. L. et al., 2021; Valikeserlis et al., 2021). MPTP is a crucial determinant of cellular damage that occurs after ischemic myocardial reperfusion (Heusch et al., 2010). The irreversible opening of this channel leads to mitochondrial collapse, further aggravating ATP depletion and cellular damage (Szabó and Zoratti, 1991; Kim et al., 2003). Myocardial ischemia deteriorates with the ensuing inflammatory response after reperfusion (Frank et al., 2012). However, timely and effective myocardial reperfusion still represents the most effective clinical treatment for myocardial ischemia. To reduce the risk of reperfusion, various strategies have emerged, including ischemic preconditioning (Arriel et al., 2020), ischemic postconditioning (Heusch, 2015), remote ischemic preconditioning, remote ischemic postconditioning (Donato et al., 2017), and therapeutic hypothermia (Sobczyk et al., 2020). These cardioprotective strategies were proven to be effective for the prevention and treatment of myocardial I/R injury in non-diabetic subjects (Mokhtari-Zaer et al., 2018; Li et al., 2022). In general, myocardial I/R injury has been extensively studied, but in patients with comorbidities, especially with diabetes mellitus, it needs to be further investigated.

## 3 Myocardial I/R injury in diabetes

Unfortunately, most strategies for the protection against myocardial I/R injury appear to be ineffective in diabetic patients (Gao et al., 2016; Lejay et al., 2016). The non-responsiveness seems to be related to the alterations in several signaling pathways (Wang et al., 2013; Gao et al., 2021). Reperfusion injury signaling kinase (RISK) pathway including PI3K/Akt signaling cascade, and survivor activating factor enhancement (SAFE) pathway including JAK2/STAT3 signaling cascade are key for myocardial protection. Defects in these pathways reduce the sensitivity of diabetic myocardium to treatments. Hyperglycemia induces the expression of phosphatase and tensin homolog deleted on chromosome ten (PTEN) and blocks PI3K/Akt pathway (Mocanu and Yellon, 2007), leading to a failure of GSK-3β phosphorylation, which promotes mPTP opening and exacerbates myocardial I/R injury (Lejay et al., 2016). Mitochondrial dysfunction is an essential cause of irreversible myocardial damage. STAT3, an effector in the SAFE pathway, is down-regulated in diabetes, leading to impairment of mitochondrial function (Wang et al., 2013). Persistent hyperglycemia of diabetes can also disrupt mitochondria by increasing dynamin-related protein 1 (DRP1) expression (Ding et al., 2017), blocking the mitochondrial K_ATP_ channel (Li et al., 2013), and inactivating hypoxia-inducible factor-1 (HIF-1α) (Riquelme et al., 2016). The above factors are highly integrated and contribute to the desensitization of diabetic myocardium to therapeutic interventions against I/R injury.

## 4 Dexmedetomidine and its potential protection against myocardial I/R injury

### 4.1 Dexmedetomidine and its potential preconditioning

#### 4.1.1 Dexmedetomidine preconditioning induced cardioprotection against non-diabetic I/R injury

Rational use of DEX for preconditioning is effective in reducing the damage caused by myocardial I/R. It was verified in numerous animal studies that cardioprotection of DEX preconditioning could be achieved through the administration of DEX (10 nM) intravenously 30 minutes before ischemia (Okada et al., 2007; He et al., 2019). In rats model of myocardial I/R injury, DEX preconditioning could reduce myocardial infarct size, decrease the incidence of arrhythmia and improve left ventricular function (Dong et al., 2017; He et al., 2019; Xiong et al., 2021). Specific mechanisms of its cardioprotective effects were explored. It was revealed that DEX might protect the heart through its direct effects on myocardial signaling cascades, such as activation of the RISK, in particular, the PI3K/Akt signaling pathway (Zhang et al., 2020c).

DEX has been recommended to be used as an auxiliary sedative for cardiac patients. For those who underwent cardiac surgery with cardiopulmonary bypass (CPB), a loading dose of 1 μg/kg DEX with a continued infusion at 0.5 μg/kg/h could reduce intraoperative myocardial injury and attenuate inflammatory responses and oxidative stress, as evidenced by decreases in CK-MB, inflammatory factors and MDA (Bayram et al., 2014; Chen et al., 2021). A retrospective study of 2,068 cardiac surgery patients showed that DEX pretreatment was associated with a better cardiac outcome and a 7% increase in 5-year patient survival (Peng et al., 2021) (Table 1). Elucidating the molecular mechanism underlying the cardioprotection benefits of DEX would be useful for finding new promising pharmacological targets and catalyze the translation of cardioprotection related research into clinical settings.

**TABLE 1:** Summary of clinical applications related to DEX.

Interventions	Main results	References
Intravenous DEX infusion of 1.0 μg/kg for 10 min prior to anesthesia, then 0.5 μg/kg/h DEX for maintenance	Preventing possible renal injury from cardiac angiography in pediatric patients by decreasing plasma endothelin-1 and renin	Bayram et al. (2014)
Intravenous DEX infusion of 0.007 μg/kg/min was initiated before or immediately after cardiopulmonary bypass and lasted for <24 h	Improving 5-year survival in patients undergoing cardiac surgery	Peng et al. (2021)
Intravenous DEX infusion of 1.0 μg/kg for 10 min prior to anesthesia, then 0.5 μg/kg/h DEX for maintenance	Reducing myocardial injury, inhibiting the release of inflammatory factors, promoting the release of anti-inflammatory factors, enhancing the activity of antioxidant enzymes and reducing oxidative stress and stress responses	Chen et al. (2021)
Intravenous DEX infusion of 0.4–0.8 μg/kg/h for maintenance	Maintaining blood glucose levels at a constant level relative to baseline in diabetic patients within 24 h postoperatively	Hui Yun and Suk Choi, (2016)
DEX as an adjuvant to spinal anesthesia	Stabilizing hemodynamics	Ye et al. (2021)

DEX, dexmedetomidine.

#### 4.1.2 Dexmedetomidine preconditioning induced cardioprotection against diabetic I/R injury

By stabilizing and maintaining blood lipids and blood glucose levels in patients with type 2 diabetes mellitus, DEX preconditioning is expected to be a new treatment modality for diabetic myocardial I/R injury (Hui Yun and Suk Choi, 2016; Sun et al., 2019). Arslan et al. (2014) proposed that DEX could improve the deformability of erythrocytes in rats model of diabetic myocardial I/R injury. By maintaining the blood glucose level and reducing oxidative stress-induced damage to cell membrane, DEX preconditioning could attenuate the alteration of erythrocyte deformability (Ozer et al., 2018), improve microcirculation and tissue perfusion (Simchon et al., 1987; Mokken et al., 1992), and offer protection against diabetic myocardial I/R injury. Several recent studies demonstrated that DEX preconditioning had cardioprotective effects against I/R injury in both streptozotocin-induced type 1 diabetic rats (Chang et al., 2020) and type 2 diabetic rats (Deng et al., 2019; Li et al., 2019; Guo et al., 2020) as evidenced by significantly reduced myocardial infarct sizes and plasma cTnT levels.

DEX also confers protection against remote organ injury caused by diabetic myocardial I/R insult. Intraperitoneal injection of DEX protected against myocardial I/R induced lung damage in diabetic rats, as evidenced by decreased neutrophil infiltration/aggregation and lung injury scores (Kip et al., 2015). More studies are needed to investigate the effect of DEX on different organ injuries resulting from diabetic myocardial I/R, which may contribute to the understanding of the underlying mechanisms of tissue damage associated with I/R injury in diabetes. The above studies revealed the unique features of DEX in diabetic myocardial I/R injury, which are the ability of DEX to balance blood glucose and lipids at a certain level, minimize the damage to the circulatory system, and mitigate injuries and adverse effects on distal organs.

Generalizing and exploring the dosage of DEX used has important implications for studying its effect on myocardial protection. We summarized the common doses of DEX used in most experimental studies. It seemed that a large dose of DEX usage was more likely to be beneficial than detrimental. The dosage of DEX used in animal models ranges from 1 μg/kg to 100 μg/kg, and 1 µM for mostly cellular models (Tables 2, 3) (Kip et al., 2015; Oh et al., 2019; Zhang et al., 2020b). It is necessary to determine the exact pharmacological dose of DEX that is biologically effective or harmful so that the side effects of DEX such as hypotension and cardiac arrest can be effectively avoided. In humans, DEX is administered as a premedication at a dose range of 0.33–0.67 μg/kg (Afonso and Reis, 2012). The above studies give a reference for an appropriate use of DEX in diabetic myocardial protection. Nevertheless, the cardioprotective effect of DEX preconditioning on I/R injury in diabetic patients has rarely been reported. One possible explanation is that the diabetic myocardium is highly vulnerable to I/R injury and the effect of DEX preconditioning is quite limited. DEX’s potential of lowering blood pressure and causing bradycardia or even sinus arrest might also compromise its cardioprotective effects (Reel and Maani, 2022).

**TABLE 2:** Molecular mechanisms of DEX in the treatment of diabetic myocardial I/R injury at the animal level.

	Interventions	Main results	References
DEX Preconditioning	Intravenously injected with DEX at a rate of 1 μg/kg/h for 28 days	Attenuating autophagy *via* the regulation of ERK and Akt signaling and improving cardiac malfunction	Oh et al. (2019)
	Intravenously injected with 7.5 μg/kg DEX at a rate of 5 μg/kg/h, 30 min before surgery	Alleviating cardiomyocyte apoptosis by mitigating myocardial endoplasmic reticulum stress	Li et al. (2019)
	Intraperitoneally injected with100 μg/kg DEX, 30 min before the ischemia period	Improving the deformability of erythrocytes and improving microcirculation	Ozer et al. (2018); Arslan et al. (2014)
	Intraperitoneally injected with100 μg/kg and 10 μg/kg DEX, 30 min before the ischemia period	Inhibiting cell apoptosis and oxidative stress by activating the PI3K/Akt pathway	Chang et al. (2020)
	Intraperitoneally injected with100 μg/kg DEX, 30 min before the ischemia period	Decreasing lung injury following myocardial I/R	Kip et al. (2015)
	Intravenously injected with 1 μg/kg DEX, and then 15 min administration of 0.7 μg/kg/h DEX	Reducing the cTnT levels, the post-reperfusion arrhythmia score and the infarct size by the induction of GSK-3β phosphorylation	Deng et al. (2019)
	Perfusion of 3 nM DEX over 5 min, followed by a 5-min wash-out period before 33 min of ischemia	Reducing myocardial infarct size and improving cardiac function	Torregroza et al. (2020)
	Perfusion of 10 nM DEX, 25 min before ischemia	Inhibiting inflammation *via* TLR4/MyD88/NF-κB pathway	Yang et al. (2017)
	Intravenously injected with 6 μg/kg DEX, and then 15 min administration of 0.7 μg/kg/h DEX	Inhibiting inflammation *via* TLR4/MyD88/NF-κB pathway	Zhang et al. (2017)
	Intracoronary infusion of DEX at a rate of 1 ng/ml, 10 ng/ml, or 100 ng/ml	Exerting the protective effect by regulating the autonomic nervous system	Yoshitomi et al. (2012)
DEX Postconditioning	Intravenously injected with 10 μg/kg DEX 5 min before reperfusion and then subjected to 120 min of reperfusion	Inhibiting oxidative stress and apoptosis *via* PI3K/Akt pathway	Cheng et al. (2018)
	Intravenously injected with 10 μg/kg DEX	Decreasing overautophagy *via* Sirt1/mTOR pathway	Ding et al. (2015); Zhang et al. (2020B)

DEX, dexmedetomidine; GSK-3β, glycogen synthase kinase-3β; I/R, ischemia/reperfusion; NF-κB, nuclear factor κB; ERK, extracellular signal-regulated kinase; PI3K, phosphatidylinositol 3-kinase; Akt (PKB), protein kinase B; SIRT1, silent information regulator 1; TLR4, toll-like receptor 4; mTOR, mammalian target of rapamycin.

**TABLE 3:** Molecular mechanisms of DEX in the treatment of diabetic myocardial I/R injury at the cellular level.

	Interventions	Main results	References
DEX Preconditioning	H9c2 cardiomyocytes were treated with DEX (1 μM) for 12 h before H/R	Inhibition of ERS-dependent apoptosis *via* CHOP signaling pathway	Li et al. (2019)
	Cardiomyocytes were treated with DEX for 1 h	Attenuation of OGD/R-induced apoptosis in cardiomyocytes by activating the PI3K/Akt pathway	Chang et al. (2020)
	H9c2 cardiomyocytes were treated with DEX (1 μM) for 1 h before hypoxia	Inhibition of ERS *via* Sirt1/CHOP pathway	Ding et al. (2015); Zhang et al. (2021)
	H9c2 cardiomyocytes were treated with DEX until the final concentration reached 5 μmol/L	Up-regulation of autophagy	Shi et al. (2021)

DEX, dexmedetomidine; OGD/R, oxygen-glucose deprivation and reoxygenation; H/R, hypoxia/reoxygenation; PI3K, phosphatidylinositol 3-kinase; Akt (PKB), protein kinase B; CHOP, C/EBP, homologous protein; ERS, endoplasmic reticulum stress; Sirt1, silent information regulator 1.

#### 4.1.3 Protection mechanisms of dexmedetomidine preconditioning in diabetic I/R injury

By inhibiting GSK-3β and affecting oxidative stress, apoptosis, calcium overload, and mPTP opening, DEX preconditioning presumably shields the myocardium from diabetes-induced activation of GSK-3β-mediated pathogenic effects (Chang et al., 2020; Guo et al., 2020). Another molecule possibly regulated by DEX is Sirt1, the absence of which is also closely related to the resistance of diabetic myocardium to medical interventions against I/R injury (Ding et al., 2015; Zhang Y. et al., 2021). In addition, the autonomic nervous system may also be involved.

##### 4.1.3.1 Dexmedetomidine preconditioning and oxidative stress, apoptosis

DEX grants an anti-diabetic myocardial I/R injury profile through anti-apoptosis and anti-oxidant stress pathways (Chang et al., 2020). Preconditioning of H9c2 cardiomyocytes with DEX significantly mitigated apoptosis and oxidative stress induced by hyperglycemic hypoxic/reoxygenation injury (Chang et al., 2020). The protective effects of DEX against diabetic I/R injury-induced apoptosis and oxidative stress in cardiomyocytes might be mediated by inhibition of PI3K/Akt pathway (Chang et al., 2020). GSK-3β plays an important role in necrosis and apoptosis of cardiomyocytes as one of the downstream targets of PI3K/Akt pathway (Miura and Miki, 2009). GSK-3β activity is a determinant of the threshold for mPTP’s opening in cardiomyocytes (Yin et al., 2012). Its phosphorylation or inactivation can inhibit the opening of mPTP, which is associated with the upregulation of apoptotic cascade response process and oxidative stress (Yin et al., 2012). In the diabetic state, GSK-3β is activated and its activity is 2-fold higher than in non-diabetic state (Yin et al., 2012). DEX preconditioning could promote the phosphorylation of GSK-3β, inhibit mPTP opening, maintain mitochondrial function, block apoptotic cascade initiation, and enhance myocardial antioxidant defense (Kip et al., 2015; Guo et al., 2020). Intraperitoneal injection of DEX attenuates ischemia-reperfusion injury by reducing the upregulated expression of apoptosis-associated protein (p-BAD, BAX) and oxidative stress-related protein (MAD) in diabetic heart (Chang et al., 2020). Hyperglycemia dramatically induced cardiac dysfunction and ultrastructural disruption following I/R injury. Treatment with DEX remarkably ameliorated these abnormalities. Yohimbine, an α2-adrenergic receptor antagonist, could block the cardioprotective effects induced by DEX by inhibition the phosphorylation of GSK-3β (Deng et al., 2019). The underlying mechanisms that involved in the negative modulation of cardiomyocyte apoptosis and oxidative stress in these studies further proved the protective role of DEX in diabetic I/R-induced cardiac injury. However, in the current study, the molecular mechanism of DEX to attenuate apoptosis and oxidative stress induced by diabetic myocardial I/R is not sufficient, and further studies are needed to be carried out.

##### 4.1.3.2 Dexmedetomidine preconditioning and endoplasmic reticulum stress

The endoplasmic reticulum is the arena where proteins are folded, modified and processed. Its normal function is crucial to the stability of the intracellular environment (Yan et al., 2019). The significant role of endoplasmic reticulum stress (ERS) in the pathogenesis of diabetic myocardial I/R injury has been widely accepted (Li W. et al., 2020). Inhibition of ERS would be an effective strategy to treat diabetic myocardial I/R injury. The myocardial protection of DEX in diabetes is associated with ERS inhibition. Preconditioning H9c2 cardiomyocytes with DEX (1 μM) could decrease the expression of mitochondrial apoptotic proteins and ERS-related proteins, including glucose-regulated protein (GRP78), C/EBP-homologous protein (CHOP), ERO1α, ERO1β and PDI. ERS agonist reversed the effects of DEX on hypoxia/reoxygenation (H/R)-induced apoptosis (Li W. et al., 2020). In diabetic rats model of myocardial I/R injury, DEX preconditioning could inhibit diabetes-exacerbated ERS and significantly reduce myocardial infarct size and improve myocardial ultrastructure damage (Li et al., 2019). The above results suggest that DEX is a novel myocardial protective agent for the treatment of diabetic myocardial I/R injury due to its strong inhibition of ERS-dependent apoptosis pathway.

##### 4.1.3.3 Dexmedetomidine preconditioning and autophagy

Autophagy is a process of self-digestion, which can remove damaged cells and renew dysfunctional organelles and proteins (Huang et al., 2020). During myocardial I/R injury, autophagy plays diverse roles in different stages. At the stage of ischemia, autophagy is beneficial for providing cells with the energy needed and inhibiting apoptosis and necrosis. Nevertheless, at the stage of reperfusion, excessive autophagy has the detrimental effect of destroying cellular components and causing myocardial injury (Shi et al., 2019). Modulation of autophagy has been considered an appropriate therapeutic option for cardioprotection (Liu et al., 2017; Liu R. et al., 2018; Li Y. et al., 2020). Studies revealed that DEX could upregulate autophagy and reduce myocardial H/R injury in isolated cardiomyocytes under high glucose conditions (Shi et al., 2021). It was found that DEX could ameliorate cardiac dysfunction and autophagic impairment in diabetic rats by suppressing the expression of LC3B and autophagy related genes (ATG) and proteins (Oh et al., 2019). Thus it is hypothesized that DEX could exert different effects on autophagy at different stages of I/R. The autophagy regulation of DEX in diabetic myocardial I/R injury and its molecular mechanism are to be further explored in the future.

##### 4.1.3.4 Dexmedetomidine preconditioning and inflammation

Inflammation is one of the most important pathological mechanisms of myocardial I/R injury. DEX reduced the expression of inflammatory factors such as TNF-α and IL-1β, and inhibited the inflammatory response *in vivo* (Yang et al., 2017; Zhang et al., 2017). TLR4/MyD88/NF-κB signaling pathway plays a key role in the regulation of inflammation. DEX preconditioning could down-regulate HMGB1 mediated the TLR4/MyD88/NF-кB signaling pathway and attenuate myocardial I/R injury (Yang et al., 2017; Zhang et al., 2017). It is believed that the inhibition of HMGB1-mediated TLR4/MyD88/NF-κB signaling pathway may be one of the anti-inflammatory mechanisms of DEX to induce myocardial protection (Yang et al., 2017; Zhang et al., 2017). Interestingly, DEX could also reduce systemic inflammatory response through TLR4/MyD88/NF-κB pathway in lower limb surgery of diabetic patients. However, whether DEX could alleviate myocardial I/R injury in diabetes mellitus by inhibiting HMGB1 mediated inflammation remains unclear.

##### 4.1.3.5 Dexmedetomidine preconditioning and autonomic nervous system

DEX has been found to protect against myocardial I/R injury by regulating the autonomic nervous system (Yoshitomi et al., 2012). Through inhibiting the norepinephrine neuron activity in the locus coeruleus, DEX could suppress sympathetic excitation, reduce catecholamine level in the blood, and decrease cardiac load and myocardial oxygen consumption. Meanwhile, with prolonged time of diastolic perfusion and increased left ventricular coronary blood flow, the release of cardiac lactic acid was reduced and the resistance of myocardial to ischemia and hypoxia was enhanced. In addition, DEX could directly inhibit the release of cardiac norepinephrine and lower the incidence of arrhythmia in high-risk patients. The mechanism may be due to the parasympathetic effect of DEX on calcium ions transport in cardiomyocytes (Yoshitomi et al., 2012; Bao and Tang, 2020). Furthermore, studies have shown that DEX increases vagal nerve tone and triggers its anti-inflammatory effect, which may contribute to the alleviation of myocardial I/R injury (Zi et al., 2019; Ju et al., 2020). Heart rate variability reflects the balance of the patient’s autonomic nervous system. In the diabetic state, DEX affects heart rate variability by regulating autonomic nerve function (Ye et al., 2021). However, the mechanism of how DEX regulates diabetic myocardial I/R injury through autonomic nervous system has not yet been well studied.

Sirt1 is a multifunctional molecule involved in myocardial I/R injury (Tian et al., 2019; Wang et al., 2020; Wang and Hu, 2020). Overexpression of Sirt1 could improve cardiac function and protect the myocardium in diabetic rats (Ding et al., 2015). DEX inhibits oxidative stress, inflammation and apoptosis by up-regulating the expression of Sirt1 and improves myocardial I/R injury in non-diabetic states (Zhang Y. et al., 2021). The effect of DEX on Sirt1 in diabetic myocardial I/R injury is well worth studying, which will provide a new theoretical basis for the treatment of diabetic myocardial I/R injury.

Taken together, the pathophysiologic process of diabetic myocardial I/R injury is extremely complex and multifactorial. DEX can work on multiple pathways simultaneously to effectively protect diabetic myocardium against I/R damage, which might be an explanation for the effectiveness of DEX for the treatment of diabetic myocardial I/R. Hence, more powerful multi-targets drug development could be a direction for future research, which will make the therapy against diabetic myocardial I/R injury more effective.

### 4.2 Dexmedetomidine and its potential postconditioning

Pharmacological postconditioning with DEX shows more clinical advantages than invasive ischemic postconditioning (Wu et al., 2021). From the perspective of clinical application value, pharmacological postconditioning would be a more suitable alternative treatment. Results from several experimental studies seemed to be consistent with the findings that postconditioning with DEX could ameliorate myocardial I/R injury (Cheng et al., 2018; Zhang et al., 2020b). The effect of DEX postconditioning was concentration-dependent, in ranges between 0.3 and 3 nM. Increased concentrations of above 3 nM failed to further enhance the effect. The cardioprotective effect is independent of the time point and the length of application in the reperfusion period (Cheng et al., 2016; Bunte et al., 2020).

#### 4.2.1 Dexmedetomidine postconditioning-induced cardioprotection against diabetic I/R injury

The currently available data on the application of DEX postconditioning in diabetic myocardial I/R injury are minimal. A study by Cheng et al. (Cheng et al., 2018) reported that DEX postconditioning exerted the same effect as DEX preconditioning in diabetic myocardial I/R model. The potency of DEX postconditioning mediated cardioprotection was reflected in the reduction of plasma CK-MB, LDH and MDA, and an improvement in myocardial histology. However, an opposite result was found by Torregroza et al. who concluded that DEX postconditioning was unable to maintain its cardioprotective properties with acute hyperglycemia (Torregroza et al., 2020). This discrepancy was probably due to the difference in experimental models. Chen et al. investigated type 2 diabetes, a chronic inflammatory condition, whereas Torregroza et al. studied an acute hyperglycemic model. Due to the unpredictability of preconditioning, postconditioning is of higher value. Diabetes mellitus and acute hyperglycemia ought to be involved in future translational research with DEX postconditioning.

#### 4.2.2 Protection mechanisms of dexmedetomidine postconditioning in diabetic I/R injury

Similar to DEX preconditioning, the protective role of DEX postconditioning in diabetic myocardium is also associated with GSK-3β. DEX postconditioning promotes the phosphorylation of GSK-3β and inhibits its activity *via* PI3K/Akt pathway (Cheng et al., 2018; Del'Guidice and Beaulieu, 2010). The phosphorylation of GSK-3β could reduce I/R damage and protect the myocardium by adjusting the Bcl-2/Bax ratio and inhibiting the caspase-controlled apoptotic pathway (Deng et al., 2019; Wang et al., 2019; Liu et al., 2020; Sharma et al., 2020). In diabetic rats subjected to myocardial I/R, DEX postconditioning inhibited cardiomyocyte apoptosis and oxidative stress with an elevation of p-PI3K, p-Akt, and a reduction in GSK-3β. And the effects were abrogated by PI3K inhibitors (Cheng et al., 2018). In addition, a study showed that DEX postconditioning could alleviate myocardial I/R injury by activating the Sirt1/mTOR axis (Zhang et al., 2020b). Whether the Sirt1/mTOR axis plays a role in alleviating myocardial I/R injury in diabetes by DEX postconditioning has not been reported. These results suggested that both preconditioning and postconditioning of DEX activate the PI3K/Akt signaling pathway, inducing the phosphorylation of downstream kinases to inhibit several pro-apoptotic factors and the irreversible opening of mPTP, then with increased the expression of p-GSK-3β in myocardial tissue with diabetic I/R injury, effectively inhibiting apoptosis and oxidative stress. However, the degree of activation of related signaling pathway by the two conditioning methods might be different. Study has shown that acute hyperglycemia abolished the protective effect of DEX postconditioning but retained the beneficial effect of DEX preconditioning on I/R injured myocardium (Torregroza et al., 2020). This can be explained that the degree of activation of related signaling pathway by the two conditioning methods might be different. Preconditioning of DEX probably could be more effective in activation of PI3K/Akt than postconditioning of DEX. There is also a possibility that preconditioning could provide more powerful protection effect through some other unknown pathways, which needs to be further investigated.

## 5 Conclusion and perspective

**FIGURE 1:**
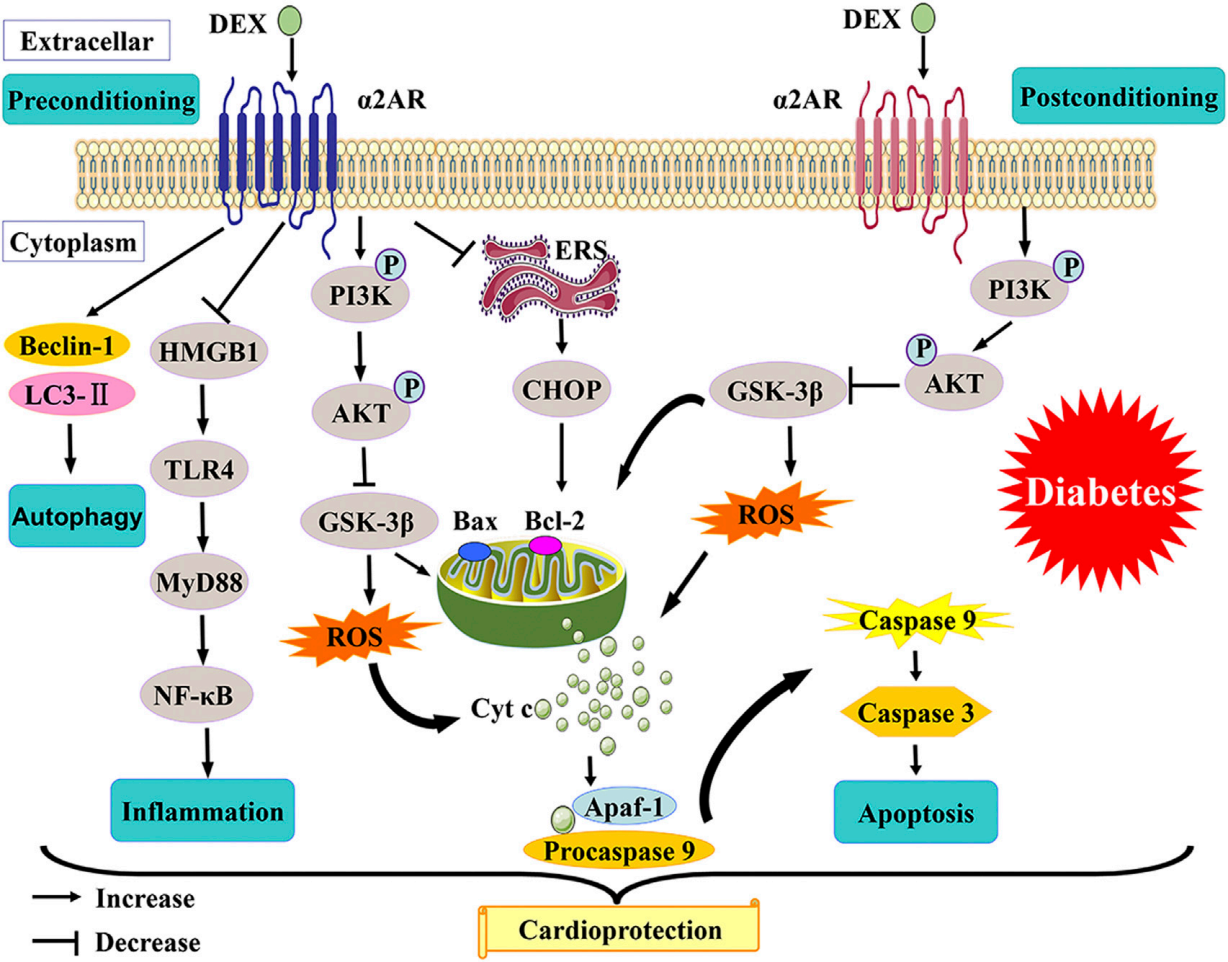
A schematic of proposed mechanisms of cardioprotection from DEX preconditioning and postconditioning in diabetes.

In this review, the evidence and the possible mechanisms of DEX in reducing myocardial I/R injury in diabetes are discussed (Figure 1). The Activation of PI3K/Akt/GSK-3β, inhibition of CHOP, attenuation of oxidative stress, regulation of autophagy, and protection of mitochondrial function are possible mechanisms that collectively contribute to DEX’s protection of diabetic myocardium. The protection of DEX on I/R insulted myocardium was not entirely dependent on the activation of α2-adrenoceptor (Yin et al., 2020). DEX may also exert protective effects by activating several other receptors (Zhang et al., 2012). This potentially interesting hypothesis needs to be verified in future studies. Other α2-adrenoceptors have not been reported in the study of diabetic myocardial protection, and their effectiveness in attenuating myocardial I/R injury in diabetes is to be confirmed. Although DEX has been widely used in various clinical scenarios, its research on diabetic myocardial I/R injury is quite limited. A better understanding of the mechanisms underlying DEX-related myocardial protection would be helpful for establishing new protective methods and developing more promising cardioprotective agents against diabetic myocardial I/R injury in the future.
